# Animal Decisions – A Look Across the Fence

**DOI:** 10.3389/fnins.2012.00142

**Published:** 2012-09-27

**Authors:** Marijn Van Wingerden, Tobias Kalenscher

**Affiliations:** ^1^Comparative Psychology, Institute for Experimental Psychology, Heinrich-Heine University DüsseldorfDüsseldorf, Germany

In this Research Topic, we have gathered some of the latest experimental results obtained in the exciting arena of studying decision-making in animals. With the dawn of the neuroeconomic method (Glimcher and Rustichini, 2004), a parallel track, or perhaps *many* parallel tracks have emerged where decision-making processes in non-human animals are being investigated. A wealth of experimental data indicates that the idiosyncrasies of human decision-making are largely mirrored in animal choice behavior, thereby both firmly establishing animal models as relevant to the study of human decision-making, and as an avenue into comparative studies of the evolution of decision-making processes. In this issue, up-to-date reviews and brand new research are mixed, featuring studies into decision-making with a cast of species spanning the animal kingdom.

Kalenscher and van Wingerden (2011) open with a review of the two points mentioned above: the similarities between humans and other animals in (deviations from) optimal choice behavior as predicted by economic theory, and the evolutionary roots of human decision-making. Highlighting differences and commonalities between species in choice behavior could help to understand the evolutionary roots of human decision-making, and perhaps help to explain why humans sometimes tend to deviate from strictly optimal choice behavior in contemporary decision-making contexts.

Next, Shizgal (2012) illustrates this comparative approach by taking a single paradigm, intracranial self-stimulation as the pursuit of a good with scarce means (operationalizing time as a handling cost). Shizgal illustrates how the behavior of the animals in this setting can be described by a component functional model, with proposed neural implementation, that incorporates the modulation of the allocation of time by reward magnitude and opportunity costs.

One has to be careful in the translation of experimental paradigms between humans and non-human animals, however, as illustrated by the report of Calvert et al. (2011). Using pigeons, these authors report that explicitly signaling the duration of common and unique delays in an intertemporal choice paradigm was sufficient to reproduce the finding in human subjects that adding a common delay to an intertemporal choice reduced the degree of discounting of the larger, later reward, whereas refraining from explicit signaling actually produced opposite results. The set of results from the explicitly signaled condition are in line with hyperbolic models of delay discounting and provide strong evidence for evolutionary conserved decision-making processes, but the other results strikingly highlight the pitfalls of assuming that a certain animal paradigm matches experimental conditions in human studies.

It has been widely recognized that most decisions are made in a social context, and that these contexts do influence decision-making. The questions remains whether this influence is restricted to humans or primates, or whether this is a more general theme in animal decision-making. Social foraging theory predicts that an animal's foraging choices are not only influenced by the balance in the rate of food intake versus the effort invested, but also by the presence of conspecifics. In two related papers, Ogura and Matsushima (2011) and Amita and Matsushima (2011)report that social manipulations in chicks did influence reward-related motivation, but not choice allocation, suggesting that at least chicks “keep their cool” in a competitive social context.

While a social context provides an example of an uncertain environment, uncertainty about future (foraging) outcomes has been traditionally operationalized as risk (uncertainty with known probabilities) and ambiguity (uncertainty about probabilities). Burke and Tobler (2011) review existing data on the neural coding of risk and ambiguity, suggesting largely independent coding of these two aspects of uncertainty that influence human and animal decision-making. Adding new experimental data, Hayden et al. (2010) contributed their latest findings on decisions under risk and ambiguity in monkeys. They show that macaques, that are usually risk-seeking display aversion to ambiguity – much like humans in a closely matched experiment with volunteers. The same authors also report the results from a single unit recording study in monkey posterior cingulate cortex (CGp, Heilbronner et al., 2011). Their results challenge the notion that this area tracks subjective value with single unit and population firing rates, as reported earlier. They show that CGp firing rates do not necessarily track the subjective value of options, as inferred from choice data, but rather are best explained by the deviation of the chosen option from a non-risky, non-delayed “standard” option across decision contexts. This deviation, which they dub “decision salience,” could be an attentional signal important for modulation of learning from outcomes. It remains to be seen whether such a neural signal exist in other species.

One of the obvious advantages to the study of non-human animals is the wider range of techniques available. Causal contributions of brain regions to decision-making can be carefully isolated and pharmacological manipulations in animals complement genetic studies involving humans suggesting involvement of neurotransmitter systems. In the clinic, one the most widely used assays for testing decision-making capacities in humans is the well-known Iowa Gambling Task. Turning to rats, de Visser et al. (2011c) reviewed four rodent gambling task models (RGTs) attempting to reproduce the canonical deficits on the Iowa Gambling Task observed by patients. The authors sketch the relative strengths and weaknesses of the four selected empirical models and discuss the translational potential of the rodent versions. In addition, de Visser and a different group of co-workers report novel experimental findings with their RGT model of choice, investigating the role of the rat medial prefrontal cortex (mPFC, de Visser et al., 2011b). They show that pharmacological mPFC inactivation seems to affect the exploitation phase of the RGT, when animals usually maintain responding on the long-term advantageous option in the face of infrequent negative outcomes, but not the exploratory phase thought to rely on a different brain regions (de Visser et al., 2011a).

It would be interesting to study simultaneous neuronal recordings from these areas as the rats proceed through the different choice strategies. However, single neuron recordings come with their own set of pitfalls. Two sets of authors discuss these pitfalls in interpreting single neuron spike data in decision-making experiments. Stüttgen et al. (2011) examined the requirements for “neurometric” data in perceptual decision-making tasks. Single neuron and population spike rates are modulated by stimuli, and usually such neurometric response curves are matched to psychometric response curves, tracking detection, choice or any other observable outcome. But identifying the “right” neurometric (rate, regularity, synchrony), as well as excluding interference between the sensation and its observable outcome are problematic. Wallis and Rich (2011) review the challenges in disambiguating decision related parameters like subjective value, saliency and motor preparation with correlational data like single unit spike recordings. To conclude the contributions, Roesch and Bryden (2011) review results from their studies of the rat single unit neural coding of reward magnitude and delay to reward, two parameters that heavily influence animal decision-making. They report that in most regions, delay to reward and reward magnitude seems to be coded by largely separate pools of neurons. In primates, it was recently shown that neural coding of risk and reward magnitude was largely separate (O'Neill and Schultz, 2010). It remains to be seen whether such decision variables, that appear to be largely integrated in compound imaging techniques such as fMRI, can be dissociated on the neural level in humans as well.

Finally, we have asked the authors of these articles to describe what, in their view, is the most important reason to investigate decision-making processes with the aid of animal experiments. Obviously, the range of techniques available for animal research is considerably larger than for human volunteers. However, even after many years of animal foraging and microeconomic research, we can still be surprised by new paradigms that show similarities between human and non-human decision-making where previously discrepancies were assumed. As mentioned by one of the contributors, and indeed also exemplified by the paper by Calvert et al. (2011), the specific context of our decision-making experiments can make all the difference in finding evidence for less or more temporal discounting, for risk-seeking or risk aversive behavior. Many authors agree that we need to think about the evolutionary context in which decision-making mechanisms evolved to appreciate their adaptive roles: comparative research across species, and thus the understanding of animal decision-making (preferably in ecologically plausible test environments) is therefore a necessary step. Other contributors mentioned that the understanding of derailed decision-making in substance abuse or psychiatric illness also relies heavily on animal research: the pharmacological irregularities found in human brains can be modeled in animals to a high degree and much of the knowledge we have of the functional networks underlying decision-making actually stems from invasive animal research. It that sense, trying to understand the animals equals trying to understand ourselves.

Besides similarities, discrepancies – sometimes large, of course remain: As one of the contributors points out, an important distinction between animal decision-making, as studied in the lab, and real-life decisions made by humans can be the uniqueness and scope of our decisions: is it at all possible to model life-changing, one-shot decisions (such as buying a house) in animals?

With this Research Topic, we hope to give an overview of the exciting research that is being carried out on animal decision-making and its relevance for the understanding of human decisions. Our hope is that researchers in the various disciplines devoted to the study of decision-making – be it economist, biologists, psychologist or other, will continue to decide to “look over the fence” every now and then. There's a world of decisions waiting to be studied.
